# How Do Profiles of Greed Map onto Multifaceted Well-Being?

**DOI:** 10.3390/bs16081286

**Published:** 2026-07-28

**Authors:** Xiaoyan Ma, Dongdong Liu, Jing Dang, Chenggang Wu

**Affiliations:** 1 Key Laboratory of Multilingual Education with AI, School of Education, Shanghai International Studies University, Shanghai 201620, China; 0253100619@shisu.edu.cn; 2 College of Teacher Education, Suqian University, Suqian 223800, China; 23179@squ.edu.cn; 3 College of Education, Inner Mongolia Normal University, Hohhot 010022, China; 20200028@imnu.edu.cn; 4 Institute of Language Sciences, Shanghai International Studies University, Shanghai 200083, China

**Keywords:** greed, well-being, life satisfaction, psychological richness, meaning in life, latent profile analysis

## Abstract

Dispositional greed is often treated as a maladaptive trait, but its links to life satisfaction, presence of meaning, search for meaning, and psychological richness, from a person-centered approach, remain underexplored. We measured these constructs in 586 Chinese university students, then used correlation analysis, latent profile analysis (LPA), and the Bolck–Croon–Hagenaars (BCH) method to test associations and profile differences. Greed correlated positively with all four indicators. LPA yielded three profiles: Low-Greed (6.7%), Average-Greed (84.0%), and High-Greed (9.4%). BCH analyses showed significant differences in psychological richness, presence of meaning, and search for meaning. The effect on life satisfaction was marginal. The Low-Greed profile scored lower than the other two profiles on psychological richness and search for meaning; the High-Greed profile also exceeded the Average-Greed profile on search for meaning. These results suggest that greed is not uniformly detrimental. Its links to well-being are graded: strongest for search for meaning and psychological richness, weakest for life satisfaction.

## 1. Introduction

The question of what constitutes a good life has long occupied both philosophy and psychology. Although well-being has traditionally been viewed through the lens of happiness and life satisfaction, recent work suggests that a fulfilling life encompasses more than positive evaluations of one’s circumstances. Individuals also seek purpose, personal growth, and experiences that broaden their perspectives. Oishi and Westgate (2022) proposed that the good life could be understood as a multidimensional construct comprising three distinct pillars: the evaluative (life satisfaction), the existential (meaning in life), and the experiential (psychological richness) (Oishi & Westgate, 2022).

Life satisfaction refers to a global cognitive evaluation of one’s overall quality of life (Diener et al., 1985). Meaning in life concerns the extent to which individuals perceive their lives as purposeful, coherent, and significant (Steger et al., 2006). Persistent dissatisfaction with one’s current circumstances may undermine these perceptions by directing attention toward what is lacking rather than what is meaningful in one’s life. Psychological richness, the most recently introduced of the three, describes a life characterized by interesting, perspective-changing, and experientially diverse events (Oishi et al., 2019; Oishi & Westgate, 2022). Although these dimensions are often positively related, they are conceptually distinct and may not always coexist. For instance, individuals may report meaningful or psychologically rich lives even when they are not highly satisfied with their circumstances. Viewing well-being through this multidimensional lens may provide a more comprehensive understanding of the psychological factors associated with a good life.

Personality traits represent one important set of correlates of well-being. Decades of research based on the Big Five framework has consistently demonstrated that traits such as extraversion and neuroticism are strongly associated with subjective well-being (DeNeve & Cooper, 1998; Steel et al., 2008). Yet the Big Five, for all their predictive utility, capture relatively broad dispositional tendencies. Broad personality dimensions may not fully capture motivational tendencies that shape how individuals pursue goals and evaluate their lives. Among these more specific motivational dispositions, dispositional greed has received increasing scholarly attention.

Dispositional greed has been defined as an insatiable desire for more and a chronic dissatisfaction with what one currently possesses (Seuntjens et al., 2015). Existing research generally portrays greed as maladaptive. Individuals high in greed tend to engage in upward social comparison, experience lower levels of satisfaction, and report greater frustration when desired outcomes are not achieved (Mussel & Hewig, 2016; Seuntjens et al., 2015). Greed has also been associated with unethical decision-making (Seuntjens et al., 2015), materialistic values (Krekels & Pandelaere, 2015), and interpersonal difficulties (Wang et al., 2011; Wang & Murnighan, 2011). Taken together, these findings have contributed to the view that greed is primarily detrimental to psychological functioning and well-being.

It is important to distinguish dispositional greed from related yet distinct constructs. Although greed shares certain features with ambition and approach-oriented tendencies, these constructs are conceptually distinct. Ambition reflects striving toward valued goals, whereas dispositional greed is characterized by a persistent sense that one never has enough regardless of actual attainment (Seuntjens et al., 2015). Accordingly, greed may be viewed as a specific motivational tendency marked by chronic wanting rather than goal pursuit itself. This distinction matters, because treating greed as a variant of ambition risks obscuring its unique association with chronic dissatisfaction and its potential to predict intensity-based well-being outcomes.

Although the predominantly negative perspective is supported by substantial evidence, it may not fully capture the complexity of greed’s associations with well-being. Most previous studies have focused on life satisfaction or general indicators of subjective well-being. Much less is known about how greed relates to other dimensions of a good life, particularly psychological richness. This limitation is important because different dimensions of well-being may be linked to personality characteristics in different ways. Because different dimensions of well-being capture different aspects of a good life, it is possible that greed may show distinct patterns of association across these dimensions. Notably, suggesting that greed may be associated with certain positive outcomes does not imply that greed is inherently adaptive. Alternative explanations should be considered. Observed associations may reflect social comparison processes, cultural norms emphasizing achievement, self-enhancement tendencies, or the possibility that greed measures partly overlap with achievement-oriented dispositions in some contexts (Paulhus & Williams, 2002). Given the cross-sectional nature of most existing studies, causal conclusions cannot be drawn. Nevertheless, these considerations suggest that the relationship between greed and well-being may be more nuanced than a uniformly negative interpretation would imply.

A further limitation of existing research concerns methodology. Most studies have employed variable-centered approaches, which focus on average relationships between variables. Although valuable, these approaches assume that individuals with similar scores on a trait are relatively homogeneous. This assumption may overlook meaningful differences in how greed is expressed across individuals. Person-centered approaches offer a complementary perspective by examining whether distinct subgroups of individuals can be identified based on their patterns of responses.

Based on the above elaboration, despite increasing interest in greed, two important gaps remain. First, research has rarely examined greed within a multidimensional well-being framework that includes psychological richness alongside life satisfaction and meaning in life. Second, little is known about whether dispositional greed exhibits meaningful latent heterogeneity and whether different greed profiles are associated with distinct patterns of well-being. Addressing these gaps may contribute to a more comprehensive understanding of the psychological correlates of greed.

Specifically, we suggest that examining greed solely through average associations with well-being may overlook important individual differences. A multidimensional and person-centered perspective may therefore provide a fuller picture of how greed relates to well-being. To address this issue, the Latent profile analysis (LPA) is a widely used person-centered method that identifies unobserved subgroups within a population (Howard & Hoffman, 2018). Rather than assuming a single pattern of association applies to all individuals, LPA allows researchers to examine whether distinct intensity-level profiles exist and whether these profiles differ in relevant outcomes. In the context of dispositional greed, a person-centered approach is particularly valuable because it can reveal whether individuals at different levels of greed exhibit distinct patterns of well-being across multiple dimensions, a question that variable-centered analyses, which assume linear and homogeneous relationships, cannot address. Applying this approach to dispositional greed may provide a more nuanced understanding of individual differences that are obscured in traditional variable-centered analyses. From this perspective, LPA offers a useful way to examine whether individuals who obtain similar overall greed scores nevertheless differ in their well-being outcomes. The present study pursued two complementary objectives. First, using a variable-centered approach, we examined the associations between dispositional greed and four dimensions of well-being: life satisfaction, presence of meaning, search for meaning, and psychological richness. Second, using a person-centered approach, we employed latent profile analysis to identify distinct greed profiles and examined whether these profiles differed across the four well-being dimensions using the Bolck–Croon–Hagenaars (BCH) method (Asparouhov & Muthén, 2014a; Bakk & Vermunt, 2016; Bolck et al., 2004).

The present study was guided by the following research questions:

RQ1: Does dispositional greed exhibit meaningful latent heterogeneity?
RQ2: Do individuals belonging to different greed profiles differ across multiple dimensions of well-being, including life satisfaction, presence of meaning, search for meaning, and psychological richness?

If greed indeed reflects chronic dissatisfaction with one’s current state, it follows logically that higher greed should predict lower life satisfaction and a diminished sense of meaning. Based on previous research linking dispositional greed to chronic dissatisfaction and lower subjective well-being, the following hypotheses were proposed.

**H1.**  *Dispositional greed will be negatively associated with life satisfaction*.

**H2.**  *Dispositional greed will be negatively associated with the presence of meaning in life*.

We deliberately did not formulate directional hypotheses for psychological richness and search for meaning due to the exploratory nature of these associations.

Clearly, this study integrates multidimensional well-being perspectives with person-centered analytical methods, facilitating a more comprehensive understanding of the complex relationship between greed and individual well-being. This would provide new empirical evidence and explanatory frameworks for relevant theoretical frameworks. Because the data are cross-sectional, we test for associations rather than causal claims, a limitation we return to in the Discussion.

## 2. Method

### 2.1. Participants

This study adopted a questionnaire survey approach, targeting emerging adults in China, with a total of 586 participants included, averaging 19.98 years of age, among whom 94 were male students. In terms of educational background, the majority of participants were undergraduates (*N* = 568), with smaller representations from high school (*n* = 9), doctoral (*n* = 4), associate degree (*n* = 3), and master’s (*n* = 2) programs. As for Grades, sophomores (*n* = 305) and juniors (*n* = 226) constituted the main group, while freshmen (*n* = 22), seniors (*n* = 27), 3rd-year doctoral students (*n* = 4), and 3rd-year master’s students (*n* = 2) were relatively few. The sample was heavily concentrated in Education (*n* = 466, 79.5%). The remaining participants were distributed across Literature (*n* = 32), Arts (*n* = 29), Law (*n* = 28), Management (*n* = 13), Science (*n* = 11), and other fields (*n* = 7; Economics, Philosophy, and Medicine). All participants signed informed consent forms prior to completing the questionnaire and retained the right to withdraw from the study at any time.

### 2.2. Measurement and Procedure

In addition to demographic information being collected, all other measurements underwent translation/back-translation procedures if there was no Chinese version available. The dispositional greed was measured by the Chinese version of dispositional greed (Liu et al., 2019), consisting of 7 items on a 7-point scale (Cronbach’s α = 0.89, CFI = 0.98, GFI = 0.97, RMSEA = 0.09, SRMR = 0.03). Well-being was operationalized as a multidimensional construct, encompassing life satisfaction, meaning in life, and psychological richness, all of which were measured on a 7-point scale. Life satisfaction was assessed using the Satisfaction With Life Scale (Diener et al., 1985), which consists of five items measuring individuals’ global cognitive evaluations of their lives (Cronbach’s α = 0.88, CFI = 0.99, GFI = 0.99, RMSEA = 0.10, SRMR = 0.02). Meaning in life was measured using the Meaning in Life Questionnaire (Steger et al., 2006), containing 10 items and including two subdimensions: presence of meaning and search for meaning (Cronbach’s α = 0.93, CFI = 0.99, GFI = 0.98, RMSEA = 0.10, SRMR = 0.02 for presence; Cronbach’s α = 0.94, CFI = 1.00, GFI = 0.99, RMSEA = 0.08, SRMR = 0.01 for search). Psychological richness was assessed with the Psychologically Rich Life Questionnaire (Oishi et al., 2019) which has 12 items (Cronbach’s α = 0.95, CFI = 0.95, GFI = 0.91, RMSEA = 0.10, SRMR = 0.04), capturing the extent to which individuals perceive their lives as interesting and experimentally diverse. Item scores were averaged to create a composite score. Only one item was reverse-coded in presence of meaning (Steger et al., 2006).

The online questionnaire, hosted on Wenjuanxing, was distributed to undergraduate students via WeChat, a widely used social media platform in China, through university-affiliated channels (e.g., class group chats). Participation was voluntary; electronic informed consent was obtained at the survey outset. No monetary or non-monetary compensation was provided.

All items were measured on a 7-point Likert scale (1 = strongly disagree, 7 = strongly agree). We computed composite scores for each scale by averaging its constituent items, with higher scores indicating higher levels of the corresponding construct.

We screened the data in three stages. First, we cross-referenced IP addresses with submission timestamps to identify duplicate entries; none were found. Second, we screened for careless responding by flagging submissions completed in under 50 s. This threshold was the minimum feasible completion time as determined through pilot testing. We also inspected response patterns for straight-lining. No responses warranted exclusion. Third, we assessed missingness across all scale and demographic variables. The core analytic variables were complete; missing data occurred only in the auxiliary “source detail” field (*n* = 140), which is not collected for WeChat submissions. Because this field was not used in any analyses, these omissions did not affect the results. All 586 responses were retained for analysis. Data were anonymized prior to analysis, and ethical research standards were maintained throughout.

### 2.3. Data Analysis Plan

All analyses were performed in JASP (version 0.97.1) and R (version 4.5.2). We began by computing descriptive statistics and Pearson correlations to inspect the distributional properties and bivariate associations among the five study variables. Cronbach’s alpha served as the index of internal consistency reliability.

Measurement validation proceeded via confirmatory factor analysis (CFA) in the lavaan package (Rosseel, 2012). Given the ordinal nature of the Likert-type items and the presence of missing data, we used robust maximum likelihood (MLR) (Satorra & Bentler, 2001) with full information maximum likelihood (FIML). The initial five-factor model was modified in two ways: item M5 was removed because its standardized loading fell below the conventional threshold and its modification index was large, and within-factor residual covariances were added sequentially based on modification indices. Model fit was judged by CFI, TLI, RMSEA, and SRMR. CFI and TLI values ≥ 0.90 and RMSEA and SRMR values ≤ 0.08 were taken as indicating acceptable fit. Convergent validity was supported by standardized factor loadings, composite reliability (CR ≥ 0.70), and average variance extracted (AVE ≥ 0.50 (Bagozzi & Yi, 1988)). We then checked discriminant validity with the Fornell–Larcker criterion (Fornell & Larcker, 1981). Because dispositional greed was modeled as a latent construct, its CFA-derived standardized factor score was carried forward as the single indicator for the subsequent LPA, thereby preserving measurement precision.

To capture both the overall linear structure and potential heterogeneity in the greed–well-being link, we adopted complementary variable-centered and person-centered strategies. At the variable level, we correlated dispositional greed with the four well-being facets: life satisfaction, psychological richness, presence of meaning in life, and search for meaning in life.

At the person level, we subjected the greed factor score to latent profile analysis (LPA) using the tidyLPA package (Rosenberg et al., 2019) with the mclust engine (Scrucca et al., 2016). Two- to four-class solutions were estimated under both equal variance–covariance (EII) and varying variance–equal covariance (VII) structures. We selected the final model by jointly inspecting AIC, BIC, SSABIC, the bootstrap likelihood ratio test (BLRT), entropy, and the relative size of the smallest class (Scrucca et al., 2016). A lower information criterion signals better fit, whereas entropy approaching 1.00 indicates sharper classification (Celeux & Soromenho, 1996). Any profile representing less than 5% of the sample was flagged as potentially unstable.

Once the optimal profile structure was established, we compared well-being across the greed-derived classes using the Bolck–Croon–Hagenaars (BCH) three-step procedure (Bolck et al., 2004; Vermunt, 2010). This was manually coded in R. The three steps included Step 1 (Classification), Step 2 (Classification Error Correction), and Step 3 (Weighted Distal Outcome Analysis). The detailed description was embedded in the Supplementary Materials.

Step 1: Classification. The measurement model was fitted to obtain posterior classification probabilities (denoted *z*) and modal class assignments (*W*) for each participant, extracted from tidyLPA output via the mclust engine. Model selection relied on BIC, AIC, sample-size-adjusted BIC (aBIC), the bootstrap likelihood ratio test (BLRT), and entropy. Lower BIC, AIC, and aBIC values indicate better relative fit, whereas higher entropy reflects clearer class separation. A significant BLRT supports the *k*-profile model over the (*k* − 1)-profile alternative. Beyond these statistical criteria, profile size, classification quality, and substantive interpretability were also weighed in the final decision.

Step 2: Classification Error Correction. The K×K classification error matrix *H* was computed next. Each element *H*_[k,t]_ gives the probability that a participant with true class membership t is assigned to class k via modal assignment. Following (Bakk et al., 2013; Vermunt, 2010):

(1) $$H_{k,t}=P(W=k|X=t)=\frac{\sum_{i=1}^{N}z_{i,t}\cdot I(W_{i}=k)}{\sum_{i=1}^{N}z_{i,t}}$$

where I(·) is the indicator function. *H* was then inverted to obtain *H*^−1^. BCH weights were generated by reweighting each participant’s posterior probabilities using the inverse of the transposed error matrix:

(2) $$w_{i,k}^{BCH}=\sum_{t=1}^{T}z_{i,t}\cdot {\left(H^{-1}\right)}_{k,t}$$

This produces an N×K matrix of individual-specific weights. Unlike simple posterior-probability weighting, the BCH correction removes first-step classification bias by explicitly accounting for classification uncertainty through matrix inversion. Negative weights occasionally emerge from sampling variability in classification error estimates (Bakk & Vermunt, 2016); these were truncated to 1 × 10^−8^.

Step 3: Weighted Distal Outcome Analysis. A weighted linear regression was fitted separately for each distal outcome. Class membership served as a categorical predictor (dummy-coded with Class 1 as reference), with BCH weights entered as observation-level weights (lm(Y ~ factor(Class), weights = w_BCH). Because the BCH procedure expands the data into long format (*K* rows per person), conventional standard errors would be too liberal. Standard errors were rendered robust to heteroskedasticity using the Huber–White HC0 estimator (Zeileis, 2004). The BCH procedure expands the data into long format (*K* rows per participant) to accommodate proportional assignment, producing unequal weights that violate the homoscedasticity assumption. This is corrected with the HC0 estimator, consistent with established BCH applications (Bakk & Vermunt, 2016; Nylund-Gibson et al., 2019).

All omnibus tests, pairwise comparisons, standard errors, and effect sizes reported in this study derive from the same corrected weighted linear regression model in Step 3. Class-specific means and SEs were taken directly from the model coefficients and the robust variance–covariance matrix. The omnibus test used a Wald F-statistic computed from these coefficients and the robust covariance matrix, with numerator df=K−1 and denominator $df=N_{c}-K$, where $N_{c}$ is the sample after listwise deletion of missing distal outcomes.

Pairwise comparisons were *t*-tests using the linear model coefficients and robust standard errors from the same model, not Welch’s *t*-tests, with $df=N_{c}-K$. Bonferroni correction controlled familywise error across all pairwise contrasts within each outcome. For each outcome, the number of pairwise comparisons was C=K(K−1)/2, yielding a Bonferroni-adjusted significance threshold of $\alpha_{adj}=\frac{0.05}{C}$. All confidence intervals for mean differences and effect sizes were constructed at the corresponding Bonferroni-adjusted confidence level $1-\alpha_{adj}$ to maintain consistency with the adjusted *p*-values. With *K* = 3 profiles, *C* = 3 and $\alpha_{adj}=0.05/3=0.0167$, corresponding to 98.33% confidence intervals.

Cohen’s *d* served as the effect size metric for all pairwise comparisons. For each class *k*, the BCH-weighted unbiased variance was computed from the weighted linear model residuals:

(3) $$s_{k}^{2}=\frac{\sum_{i=1}^{N}w_{i,k}(y_{i}-M_{k}{)}^{2}}{n_{k}^{eff}-1}$$

where *w_i,k_* is the BCH weight for person *i* in class *k*, *y_i_* is the distal outcome value, *M_k_* is the BCH-corrected mean for class *k*, and $n_{k}^{eff}=\sum_{i}w_{i,k}$ is the effective sample size for class *k*. The pooled variance for any pairwise comparison between classes *i* and *j* was then:

(4) $$s_{p}^{2}=\frac{(n_{i}^{eff}-1)\cdot s_{i}^{2}+(n_{j}^{eff}-1)\cdot s_{j}^{2}}{n_{i}^{eff}+n_{j}^{eff}-2}$$

Cohen’s *d* was calculated as the mean difference divided by the pooled standard deviation:

(5) $$d=\frac{{\bar{X}}_{i}-{\bar{X}}_{j}}{s_{p}}$$

The standard error of d followed (Hedges, 1981):

(6) $$SE(d)=\sqrt{\frac{n_{i}^{eff}+n_{j}^{eff}}{n_{i}^{eff}\cdot n_{j}^{eff}}+\frac{d^{2}}{2(n_{i}^{eff}+n_{j}^{eff})}}$$

Bonferroni-adjusted confidence intervals for Cohen’s *d* were constructed at the same $1-\alpha_{adj}$ level as the mean difference confidence intervals:

(7) $$CI=d\pm z_{\alpha_{adj}/2}\cdot SE(d)$$

where $z_{\alpha_{adj}/2}$ is the critical value from the standard normal distribution at the Bonferroni-adjusted two-tailed level. Effect size magnitudes were interpreted as negligible (|d|<0.2), small (0.2≤|d|<0.5), medium (0.5≤|d|<0.8), and large (|d|≥0.8) (please see R code in the Supplementary Materials for more details).

Uncertainty from the first-step measurement model carries over into third-step estimates, which can produce slightly underestimated standard errors if ignored (Bakk et al., 2014). The sandwich estimator handles heteroskedasticity and the complex sampling structure from proportional assignment, but it does not fully adjust for first-step estimation error. In practice, this bias is usually small when classification quality is strong and the approach aligns with current conventions in the applied literature (Bakk & Kuha, 2021; Nylund-Gibson et al., 2019).

## 3. Results

### 3.1. Descriptive Statistics and Correlation Analyses

Table 1 presents the descriptive statistics and correlation coefficients for each study variable, with the diagonal representing the square root of the average variance extract (AVE) for each latent variable. The descriptive statistics indicate that the mean values of the variables range from 3.98 to 4.60, and the standard deviations range from 1.17 to 1.27, indicating generally moderate levels with moderate dispersion.

In contrast to H1 and H2, the correlation analysis results indicate that dispositional greed correlated positively with all four well-being indicators, though the effects were modest. Correlations were small for life satisfaction (*r* = 0.112, *p* < 0.01), presence of meaning (*r* = 0.136, *p* < 0.001), and psychological richness (*r* = 0.197, *p* < 0.001), and small-to-moderate for search for meaning (*r* = 0.275, *p* < 0.001). Given the sample size (*N* = 586), all associations reached statistical significance; however, only the link with search for meaning approached a conventional moderate effect size. Life satisfaction shows strong positive correlations with psychological richness (*r* = 0.667, *p* < 0.001) and presence of meaning in life (*r* = 0.661, *p* < 0.001) while demonstrating weak correlations with search for meaning in life (*r* = 0.086, *p* < 0.05). Psychological richness exhibits a high correlation with presence of meaning in life (*r* = 0.746, *p* < 0.001) and a moderate correlation with search for meaning in life(*r* = 0.220, *p* < 0.001). Additionally, the correlation between presence of meaning in life and search for meaning in life is weak but reaches statistical significance (*r* = 0.088, *p* < 0.05).

As reported in Table 1, the square roots of the AVEs exceeded the corresponding inter-construct correlations for all constructs, supporting the discriminant validity of the measurement model.

### 3.2. Confirmatory Factor Analysis

The hypothesized measurement model was evaluated using CFA. During the model refinement process, item M5 was removed due to its relatively low standardized loading (0.34) and high modification indices. The revised five-factor model was then re-estimated. The results indicated that the measurement model demonstrated an acceptable fit to the data (χ^2^ = 1322.66, df = 482, χ^2^/df = 2.74, CFI = 0.915, TLI = 0.907, RMSEA = 0.055, SRMR = 0.050, AIC = 53,880.79, BIC = 54,370.60; see Table 2).

To examine the distinctiveness of the proposed constructs, the revised five-factor model was compared with two alternative models. In the four-factor model, presence of meaning in life and search for meaning in life were combined into a single factor. As shown in Table 2, both the four-factor (χ^2^ = 3562.40, df = 521, χ^2^/df = 6.84, CFI = 0.703, TLI = 0.680, RMSEA = 0.100, SRMR = 0.127, AIC = 56,423.10, BIC = 56,912.91, ∆χ^2^ = 529.99, Δdf = 4, *p* < 0.001) and one-factor (χ^2^ = 5679.65, df = 527, χ^2^/df = 10.78, CFI = 0.496, TLI = 0.464, RMSEA = 0.129, SRMR = 0.156, AIC = 62,635.16, BIC = 63,081.24, Δχ^2^ = 1467.40, Δdf = 10, *p* < 0.001) models demonstrated substantially poorer fit than the revised five-factor model. The significant decline in model fit for the four-factor model indicates that presence of meaning and search for meaning are better represented as separate constructs. The poor fit of the one-factor model provides some evidence against severe common method bias, although it does not entirely rule it out. We acknowledge this issue more fully in the Limitations section.

Convergent validity was assessed using standardized factor loadings, composite reliability (CR), and average variance extracted (AVE). As shown in Table 3, most standardized factor loadings exceeded 0.50, with the exception of S5 (0.449). Nevertheless, CR values exceeded 0.70 and AVE values exceeded 0.50 for all constructs. Discriminant validity was examined using the Fornell–Larcker criterion, and the square roots of the AVE were greater than the corresponding inter-construct correlations. Collectively, the results support the adequacy of the measurement model.

### 3.3. Latent Profile Analysis of Greed

To identify latent profiles of greed, models with two to four classes were estimated under EII and VII specifications. The fit statistics are reported in Table 4. A viable four-class VII solution could not be obtained. Extensive initialization attempts (nrep = 500–2000; itmax = 1000–3000; hierarchical and random starts) resulted in either convergence failure or boundary estimates, with several class-specific variances collapsing to near-zero values. This points to empirical under identification. With only one standardized indicator (the dispositional greed factor score), the data do not support freely estimating four class-specific variances, means, and mixing proportions. Reliable fit statistics (BIC, AIC, entropy) and classification metrics were therefore unavailable, and this solution is omitted. Considering model fit, profile size, and interpretability, the VII-3 solution was retained. This model yielded the lowest BIC (1719.82) and SSABIC (1694.42), and the BLRT indicated a significant improvement over the two-class solution (*p* = 0.002). The entropy value was 0.616, indicating moderate classification precision. Nevertheless, the smallest profile represented 6.7% of the sample, exceeding the recommended minimum size criterion.

**Figure 1:**
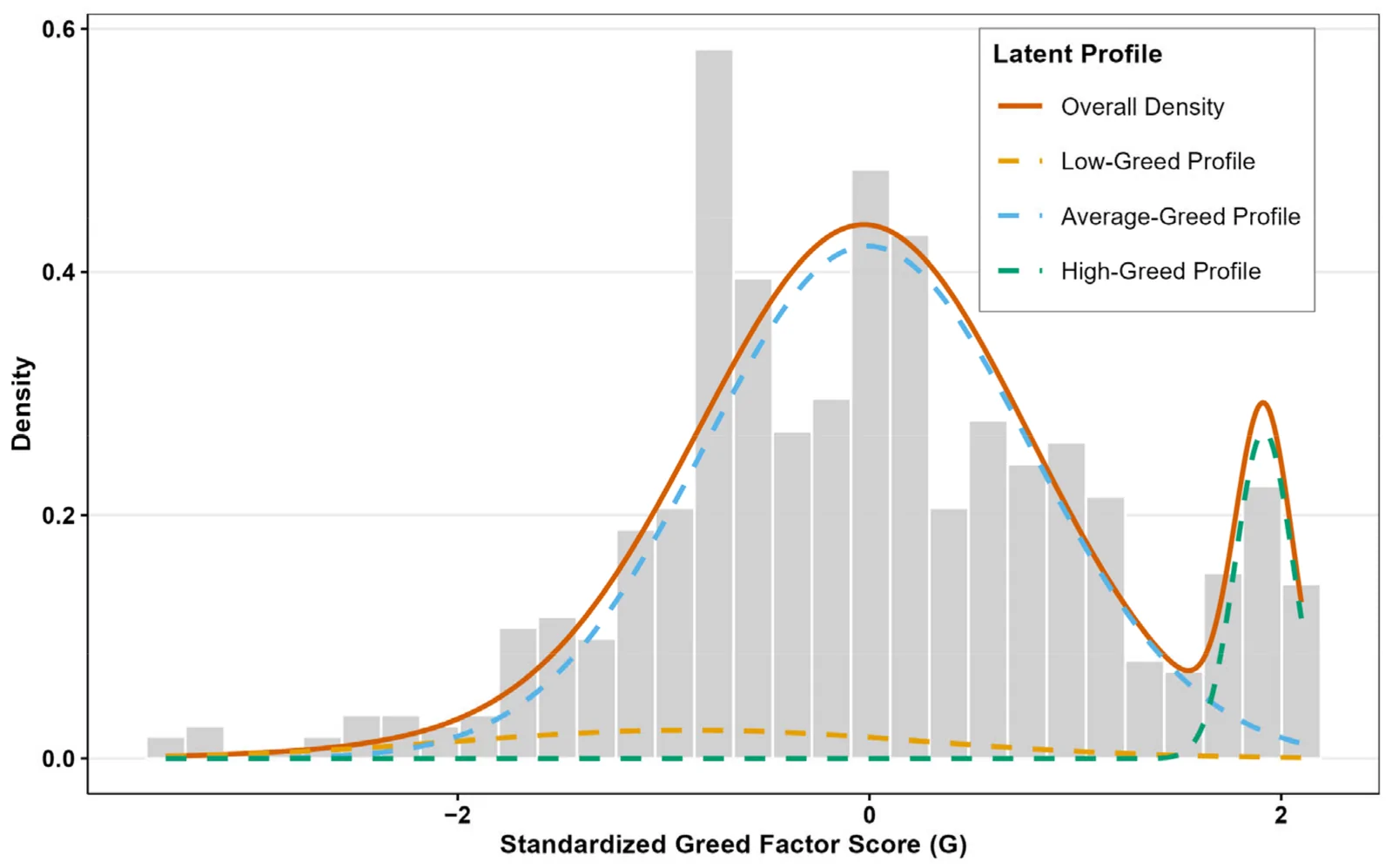
Latent profiles of different greed level segment (*N* = 586).

Table 5 and Figure 1 summarize the characteristics of the three latent profiles. Inspection of the profile means suggested three groups that differed primarily in their levels of greed. These profiles were labeled Low-Greed (*n* = 39, 6.7%, M = −0.87, SD = 1.13), Average-Greed (*n* = 492, 83.9%, M = −0.01, SD = 0.79), and High-Greed (*n* = 55, 9.4%, M = 1.92, SD = 0.14). Most participants were classified into the Average-Greed profile.

Table 6 reports the average posterior probabilities for profile membership. The probabilities associated with the Low-Greed profile, Average-Greed profile, and High-Greed profile were 0.724, 0.843, and 0.857, respectively. Overall classification accuracy was 80.8%.

### 3.4. The Relationship Between Greed and Subjective Well-Being

**Figure 2:**
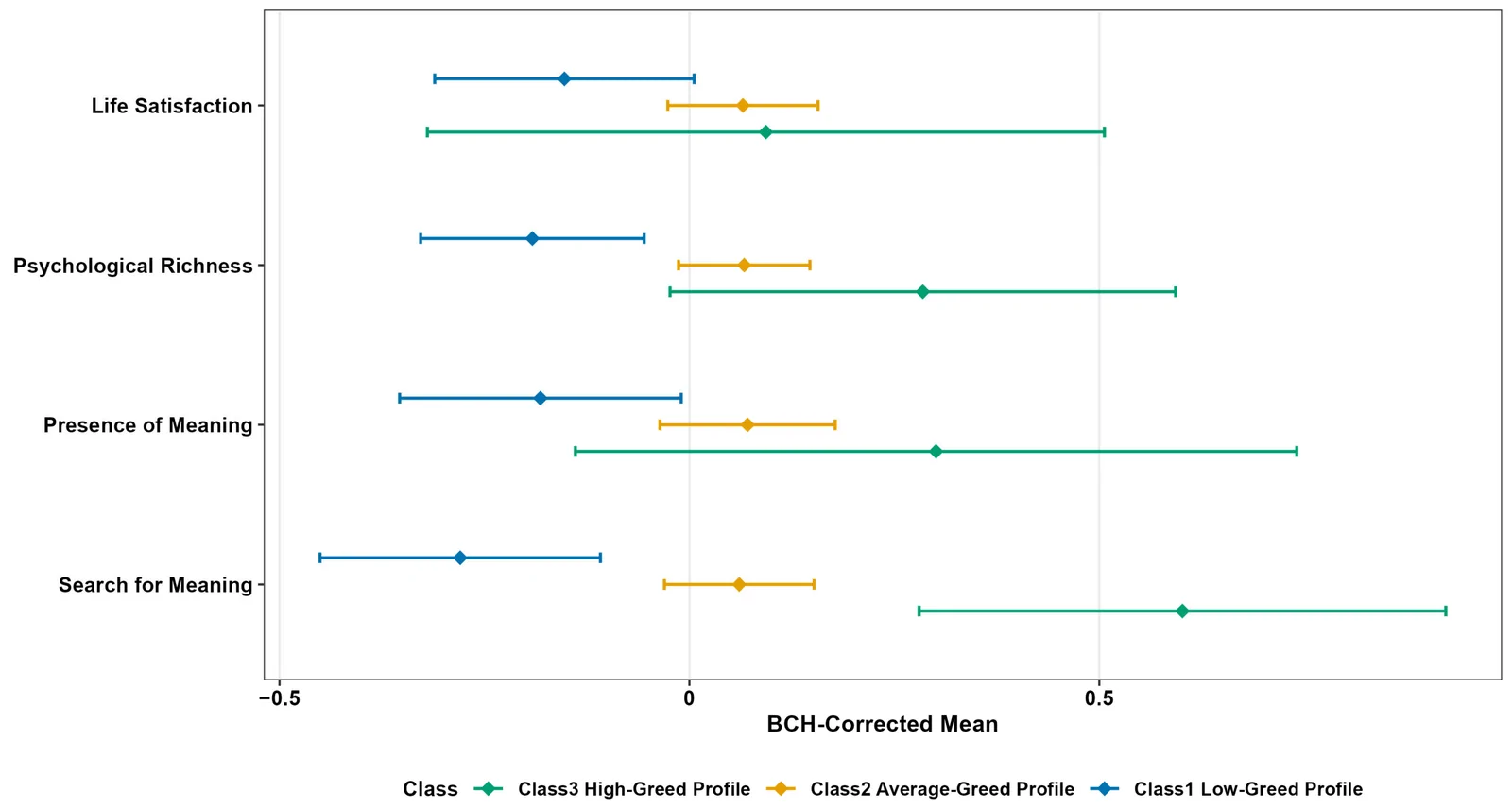
BCH-corrected distal outcome means by greed profile. Error bars represent 95% confidence intervals. *N* = 586.

Differences in subjective well-being across the three greed profiles were examined using the BCH approach. The BCH-corrected Wald *F* tests are reported in Table 7. Profile differences were significant for psychological richness (*F*(2, 583) = 6.68, *p* = 0.001), presence of meaning (*F*(2, 583) = 3.86, *p* = 0.022), and search for meaning (*F*(2, 583) = 12.57, *p* < 0.001). Life satisfaction showed a marginally significant overall effect (*F*(2, 583) = 2.80, *p* = 0.062). BCH-corrected means and 95% confidence intervals are plotted in Figure 2.

As shown in Table 8, post hoc Bonferroni-corrected pairwise comparisons were conducted. For life satisfaction, the Low-Greed group (M = −0.15, SE = 0.08) scored marginally lower than the Average-Greed group (M = 0.07, SE = 0.05), with a mean difference of −0.22, 95% CI [−0.40, −0.03], *p* = 0.060, *d* = −0.21 (small effect). The difference between the Low-Greed and High-Greed groups (M = 0.09, SE = 0.21) was not significant (*p* = 0.829).

For psychological richness, the Low-Greed group (M = −0.19, SE = 0.07) scored significantly lower than both the Average-Greed group (M = 0.07, SE = 0.04; *p* = 0.004, *d* = −0.29) and the High-Greed group (M = 0.28, SE = 0.16; *p* = 0.017, *d* = −0.48). The Average-Greed and High-Greed groups did not differ significantly (*p* = 0.542).

A similar pattern emerged for presence of meaning in life. The Low-Greed group (M = −0.18, SE = 0.09) scored significantly lower than the Average-Greed group (M = 0.07, SE = 0.05; *p* = 0.044, *d* = −0.22), but the comparison with the High-Greed group (M = 0.30, SE = 0.22) did not survive multiple comparison correction (*p* = 0.137).

Search for meaning in life demonstrated the strongest profile differences. The Low-Greed group (M = −0.28, SE = 0.09) scored significantly lower than both the Average-Greed group (M = 0.06, SE = 0.05; *p* = 0.002, *d* = −0.32) and the High-Greed group (M = 0.60, SE = 0.16; *p* < 0.001, *d* = −0.75, medium effect). Additionally, the Average-Greed group scored significantly lower than the High-Greed group (*p* = 0.005, *d* = −0.54, medium effect), revealing a clear incremental pattern.

**Figure 3:**
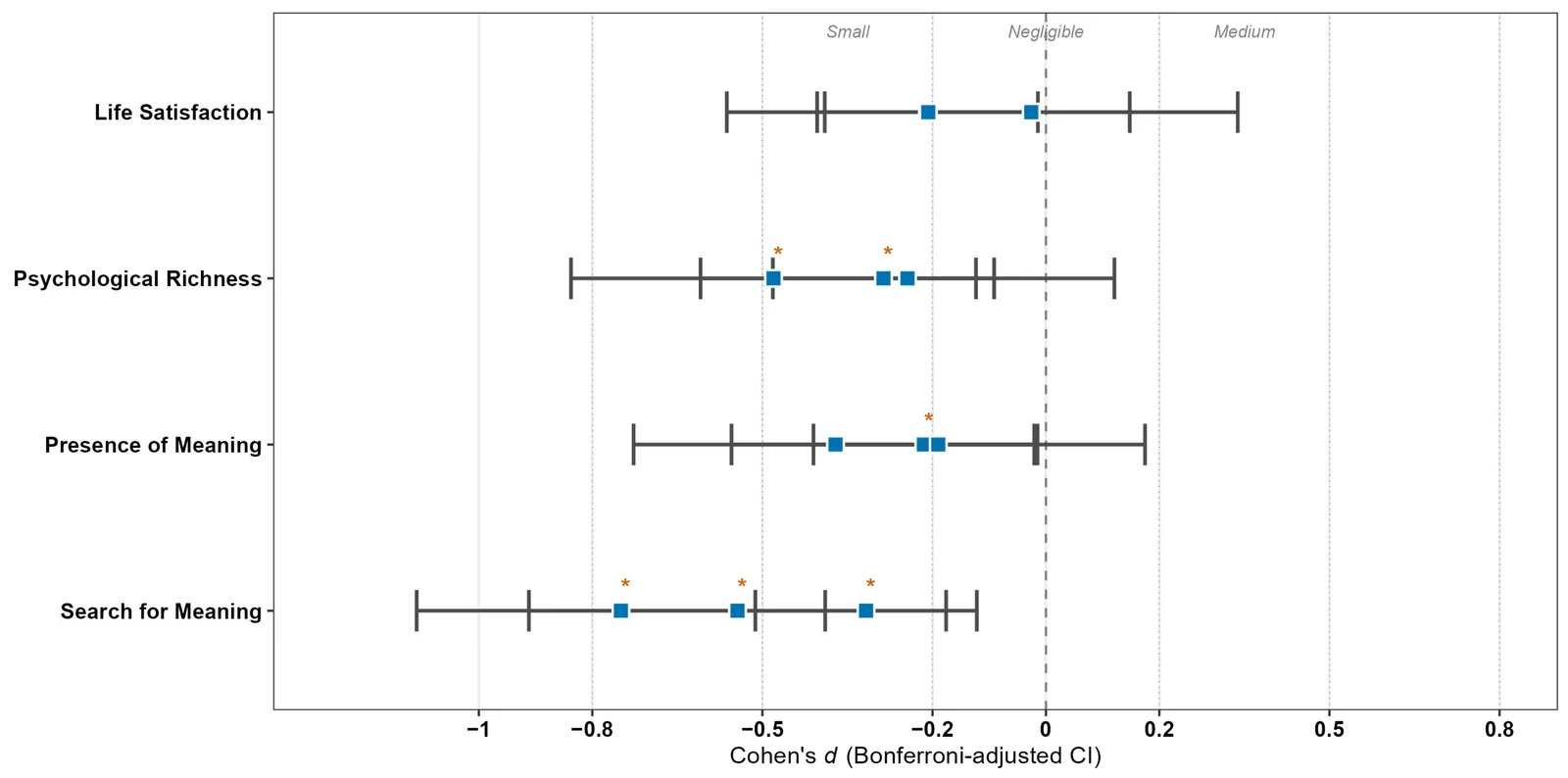
Cohen’s *d* Effect Sizes for Pairwise Class Differences on Distal Outcomes with Bonferroni-Adjusted 98.33% Confidence Intervals. *p* values were Bonferroni-corrected for three pairwise comparisons per outcome (α = 0.017). * indicates significance after correction. Reference lines denote negligible (<0.2), small (0.2–0.5), and medium (0.5–0.8) effect size benchmarks.

Effect size estimates are reported in Table 8 and visualized in Figure 3. Most significant comparisons yielded small effect sizes (|*d*| = 0.21−0.48), with only the two comparisons involving search for meaning in life reaching medium effect sizes (|*d*| = 0.54−0.75).

## 4. Discussion

### 4.1. Overview of Key Findings

The present study adopted a dual analytical approach to examine the relationship between dispositional greed and multidimensional well-being. Using a variable-centered framework, we found that dispositional greed was positively rather than negatively associated with life satisfaction, presence of meaning, search for meaning, and psychological richness. These results did not support the H1 and H2, which claimed that greed was negatively related to life satisfaction and presence of meaning. Complementing this approach, latent profile analysis (LPA) identified three intensity-based greed profiles: a small group of low-greed individuals (6.7%), a large average-greed majority (84.0%), and a high-greed minority (9.4%). BCH analyses further revealed that these profiles differed selectively across well-being dimensions. Specifically, the three greed profiles differed significantly in psychological richness (*F*(2, 583) = 6.68, *p* = 0.001), presence of meaning (*F*(2, 583) = 3.86, *p* = 0.022), and search for meaning (*F*(2, 583) = 12.57, *p* < 0.001). Life satisfaction showed a weaker, marginally significant effect (*F*(2, 583) = 2.80, *p* = 0.062). Post hoc comparisons indicated that the low-greed profile scored lower than the average-greed and high-greed profiles across these well-being dimensions. These findings suggest that the relationship between dispositional greed and well-being may be more complex than previously assumed and may vary across different dimensions of well-being.

### 4.2. Reinterpreting the Valence of Greed in Well-Being Research

Our variable-centered results stand in marked contrast to the predominantly negative portrayal of greed in the literature (Krekels & Pandelaere, 2015; Seuntjens et al., 2015; Wang et al., 2011). Rather than correlating with dissatisfaction, dispositional greed showed small to moderate positive associations with all four well-being indicators. We propose three interrelated interpretations for this unexpected pattern.

The first possibility is that the Dispositional Greed Scale (DGS) may partially capture variance associated with approach motivation and goal-directed striving (Elliot & Church, 1997). Items emphasizing the desire for “more” and the insatiability of current possessions may, in a student population, reflect achievement orientation or future-oriented ambition rather than pathological acquisitiveness. If this is the case, the observed positive correlations may index motivational intensity rather than chronic dissatisfaction per se. Second, from a functional perspective, a persistent desire for more may drive behavioral engagement, exploration, and goal pursuit. These activities directly feed into psychological richness and the search for meaning (Oishi & Westgate, 2022). Individuals who chronically want more may, somewhat paradoxically, lead more eventful and perspective-changing lives, thereby accumulating experiences that enhance psychological richness (Oishi & Westgate, 2022; Steger et al., 2006). Third, the cross-sectional nature of these associations precludes causal inference. It remains entirely plausible that individuals who experience their lives as psychologically rich or meaningful develop stronger appetites for further experiences, thereby increasing their greed scores. Longitudinal research is needed to disentangle these directional possibilities.

Notably, the strength of associations varied across well-being dimensions. The correlation was moderate for search for meaning (*r* = 0.275), small to moderate for psychological richness (*r* = 0.197), and small for life satisfaction (*r* = 0.112). This gradient suggests that greed may be more closely aligned with existential exploration and experiential diversity than with cognitive evaluations of life quality. This pattern aligns with the theoretical distinction between hedonic and eudaimonic well-being (Diener et al., 1985; Ryan & Deci, 2001; Steger et al., 2006; Waterman et al., 2008), indicating that greed may be more closely associated with eudaimonic exploration than with hedonic evaluations.

### 4.3. The Heterogeneity of Greed: Insights from the Person-Centered Approach

The latent profile analysis revealed substantial heterogeneity in the expression of dispositional greed. The three-class solution indicated that greed is not uniformly distributed but characterized by a small high-greed group, a large normative group, and a small low-greed group. Its distribution resembles a skewed normal curve. However, the LPA framework provides additional information beyond distributional characteristics by identifying subgroups that differ in the intensity of dispositional greed (Asparouhov & Muthén, 2014a; Bolck et al., 2004; Vermunt, 2010). Importantly, the three profiles appear to differ primarily in degree rather than representing fundamentally different forms of greed.

The low-greed profile (6.7%) is particularly theoretically interesting. Contrary to the assumption that “less greed” implies greater contentment, these individuals exhibited the lowest levels of psychological richness and search for meaning. This suggests that extremely low levels of dispositional greed may be associated with lower tendencies toward exploration and novelty seeking (Ravert et al., 2013), rather than necessarily reflecting greater contentment or well-being. However, because motivational processes were not directly assessed in the present study, this interpretation should be considered tentative.

In contrast, the high-greed profile (9.4%) showed elevated levels on these same dimensions. The average-greed majority, representing the statistical norm, fell between these extremes. These findings underscore the value of person-centered methods: had we relied solely on variable-centered correlations, we might have concluded that greed is positively associated with well-being across the sample, whereas the LPA reveals that the contrast between the low-greed and high-greed profiles appears particularly informative for understanding profile differences in well-being.

### 4.4. Dimension-Specific Profile Differences and Their Interpretation

The BCH analyses revealed a graded pattern of profile differences across the four well-being dimensions. Search for meaning exhibited the strongest and most consistent differences, followed by psychological richness, presence of meaning, and life satisfaction. This gradient carries important theoretical implications.

For search for meaning in life, all three pairwise comparisons were statistically significant after Bonferroni correction. The low-greed group scored lower than both the average-greed group (*d* = −0.32) and the high-greed group (*d* = −0.75), and the average-greed group scored lower than the high-greed group (*d* = −0.54), revealing a clear incremental pattern. The medium effect sizes for comparisons involving the high-greed group suggest that dispositional greed may be meaningfully associated with individual differences in the active search for purpose and direction. This finding resonates with (Steger et al., 2008) observation that the search for meaning reflects an active, dynamic orientation toward understanding one’s purpose, a disposition that may share variance with the chronic desire for more that characterizes dispositional greed.

Psychological richness also showed robust profile differences. The low-greed group scored significantly lower than both the average-greed group (*d* = −0.29) and the high-greed group (*d* = −0.48), though the latter two did not differ significantly. This pattern suggests that higher levels of dispositional greed are associated with greater experiential diversity and perspective-changing experiences (Oishi & Westgate, 2022). One possible interpretation is that individuals with higher levels of dispositional greed may be more likely to report a greater interest in novelty, opportunity, and future possibilities, which may in turn be associated with higher levels of psychological richness. Nevertheless, this interpretation remains speculative and requires direct empirical examination.

Presence of meaning showed a significant overall effect (*F*(2, 583) = 3.86, *p* = 0.022), though post hoc comparisons were more differentiated. The low-greed group scored significantly lower than the average-greed group (*d* = −0.22), but the comparison with the high-greed group did not survive Bonferroni correction (*p* = 0.137). This pattern suggests that the relationship between greed and presence of meaning is not linear across the full range of the greed continuum. Rather, the most pronounced difference lies between those with unusually low levels of greed and the statistically normative average-greed majority. Both high-greed and average-greed individuals may derive meaning from their respective lifestyles, whether through accumulation and achievement or through more conventional goal pursuit.

Life satisfaction showed only a marginally significant overall effect (*F*(2, 583) = 2.80, *p* = 0.062). The low-greed group scored marginally lower than the average-greed group (*p*_adj = 0.060, *d* = −0.21), but the comparison with the high-greed group was far from significant (*p*_adj = 0.829). This suggests that once individuals are categorized into greed profiles, the overall cognitive evaluation of life quality becomes relatively homogeneous across the average- and high-greed groups. Life satisfaction may be buffered by factors such as social comparison standards, cultural norms, or baseline affectivity that operate independently of greed-related motivational differences (Lyubomirsky & Ross, 1997; Lyubomirsky & Tucker, 1998).

Collectively, these findings reveal a clear gradient: the profile differences are biggest for search for meaning (medium effect sizes), moderate for psychological richness (small to medium effects), and weaker for presence of meaning and life satisfaction (small effects). This gradient aligns with the theoretical distinction between hedonic and eudaimonic well-being (Diener et al., 1985; Ryan & Deci, 2001; Steger et al., 2006; Waterman et al., 2008) and suggests that dispositional greed may be most closely associated with active existential exploration and experiential diversity rather than with the cognitive evaluation of life quality or the sense that one’s life is inherently meaningful.

### 4.5. Alternative Explanations

Several alternative explanations should be considered when interpreting the present findings. First, the observed associations may partly reflect achievement-oriented tendencies that overlap with dispositional greed. As noted above, some aspects of the DGS may capture striving, aspiration, or future-oriented goal pursuit rather than purely maladaptive acquisitiveness (Elliot & Church, 1997). Second, cultural factors may play an important role. Within the Chinese educational context, a desire for “more” may be interpreted less negatively and may be more closely linked to self-improvement and academic achievement (Liu et al., 2019). Third, self-enhancement and response-style biases may have contributed to the positive associations observed among self-reported variables (Podsakoff et al., 2003). Consequently, the present findings should not be interpreted as evidence that greed itself promotes well-being. Rather, they suggest that greed may be associated with certain well-being dimensions in ways that are more complex than traditionally assumed.

### 4.6. Theoretical Implications

Collectively, our findings call for a more differentiated theoretical conceptualization of dispositional greed. More broadly, they contribute to an emerging perspective that personality traits may have dimension-specific associations with well-being rather than uniformly positive or negative consequences (Disabato et al., 2016; Waterman et al., 2008). The prevailing view, which positions greed as a uniformly maladaptive trait linked to dissatisfaction and unethical behavior (Seuntjens et al., 2015, 2019; Wang et al., 2011), may be incomplete. Instead, the present findings suggest that dispositional greed may be associated with differences in motivational orientation and well-being experiences. Rather than being uniformly maladaptive, greed appears to relate differently to distinct dimensions of well-being.

Moreover, this study underscores the necessity of adopting a multidimensional well-being framework. Had we restricted our investigation to life satisfaction, a traditional hedonic indicator, we would have concluded that greed is largely irrelevant to well-being. It is only by incorporating psychological richness and the search for meaning that the motivational significance of greed becomes apparent. This aligns with (Oishi & Westgate, 2022) argument that a comprehensive understanding of the good life requires moving beyond happiness and meaning to include experiential diversity.

Finally, the convergence of variable-centered and person-centered findings strengthens our conclusions. The positive correlations in the total sample, combined with the specific profile differences identified via LPA and BCH (Asparouhov & Muthén, 2014b; Bolck et al., 2004), suggest that the relationship between greed and well-being is robust across analytical frameworks but manifests most clearly in eudaimonic and experiential dimensions.

### 4.7. Practical Implications

These findings carry tentative implications for educational and counseling contexts. Rather than uniformly discouraging greedy tendencies, the present findings suggest that practitioners might benefit from distinguishing between different forms of acquisitive motivation. Future intervention research may examine whether certain motivational tendencies associated with greed can be channeled toward constructive goals, such as learning, creativity, or personal development. However, because the current study did not directly examine intervention effects or underlying mechanisms, these implications should be considered preliminary.

### 4.8. Limitations and Directions for Future Research

Several limitations warrant caution in interpreting these findings. First, the cross-sectional design precludes causal conclusions. The observed associations may reflect reverse causality or bidirectional influences. These possibilities that can only be addressed through longitudinal designs (Steger et al., 2009). Second, all measures were self-reported, raising concerns about common method variance and social desirability (Podsakoff et al., 2003, 2012). Although the CFA results and the poor fit of the one-factor model suggest that common method bias was not a dominant threat, it cannot be entirely ruled out. Third, our sample consisted of Chinese university students, a highly educated and culturally specific population. The meaning of “greed” in Chinese contexts may carry connotations of ambition or self-improvement that differ from Western conceptualizations, potentially inflating the observed associations with well-being (Liu et al., 2019). Future research should replicate these findings in non-student, cross-cultural, and longitudinal samples.

Methodologically, the removal of item M5 during CFA model refinement, while improving fit, may have altered the construct representation of the greed measure. Future studies should examine whether this item functions differently across cultures. Additionally, the entropy value of 0.616 for the three-class solution indicates moderate classification precision; some profile misclassification is likely, which may have attenuated the BCH differences. Future research could employ larger samples to achieve more precise classification (Asparouhov & Muthén, 2014b; Bolck et al., 2004; Vermunt, 2010). Our LPA was based on a single composite score of dispositional greed. Therefore, the identified profiles primarily differentiate individuals along a continuum of greed intensity rather than revealing qualitatively distinct subtypes.

Future studies should also incorporate behavioral and experimental measures to complement self-reports. For example, experience-sampling methods (Silvia et al., 2014) could assess whether high-greed individuals actually engage in more diverse daily activities, or whether laboratory tasks reveal differential patterns of risk-taking and novelty-seeking. Finally, given the unexpected positive associations, qualitative research exploring how individuals interpret and experience their own “greed” would provide valuable phenomenological depth.

Rather than asking whether greed is inherently beneficial or harmful, future research should examine how greed interacts with different psychological processes and well-being dimensions. We tested this proposition using a multidimensional, person-centered approach. Greed and well-being were positively correlated across the full sample, but the low-greed profile scored lower than both the average-greed and high-greed profiles on psychological richness, presence of meaning, and search for meaning. These findings suggest that the observed associations between greed and well-being vary depending on how well-being is conceptualized and measured. Rather than asking whether greed is “good” or “bad” for well-being, future research should examine under what conditions, for which individuals, and in relation to which life domains greed becomes psychologically consequential.

## Figures and Tables

**Table 1:** Descriptive statistics and correlation matrix.

Variables	M	SD	1	2	3	4	5
1. Dispositional Greed	4.60	1.17	**0.727**				
2. Life Satisfaction	3.98	1.17	0.112 **	**0.781**			
3. Psychological Richness	4.36	1.20	0.197 ***	0.667 ***	**0.791**		
4. Presence of Meaning	4.25	1.27	0.136 ***	0.661 ***	0.746 ***	**0.874**	
5. Search for Meaning	4.48	1.19	0.275 ***	0.086 *	0.220 ***	0.088 *	**0.872**

Note. *N* = 586. The diagonal values in bold represent the square root of AVE. * *p* < 0.05, ** *p* < 0.01 and *** *p* < 0.001.

**Table 2:** Model Fit Comparison for Alternative CFA Models.

Model	χ^2^	df	χ^2^/df	CFI	TLI	RMSEA [90% CI]	SRMR	AIC	BIC
**Five-factor model (final)**	**1322.66**	**482**	**2.74**	**0.915**	**0.907**	**0.055 [0.052, 0.057]**	**0.050**	**53,880.79**	**54,370.60**
Five-factor model (initial)	1841.54	517	3.56	0.870	0.859	0.066 [0.064, 0.069]	0.069	56,423.10	56,912.91
Four-factor model	3562.40	521	6.84	0.703	0.680	0.100 [0.097, 0.102]	0.127	59,179.20	59,651.52
One-factor model	5679.65	527	10.78	0.496	0.464	0.129 [0.127, 0.132]	0.156	62,635.16	63,081.24

Note. *N* = 586. CFI = comparative fit index; TLI = Tucker–Lewis index; RMSEA = root mean square error of approximation; CI = confidence interval; SRMR = standardized root mean square residual; AIC = Akaike information criterion; BIC = Bayesian information criterion. The refined five-factor model excluded item MP5 to improve model fit. Bold values indicate the optimal model.

**Table 3:** Standardized Factor Loadings, Reliability, and Convergent Validity of the Five-Factor Measurement Model.

Construct	Dimensions	Items	Loading	Cronbach’s Alpha	CR	AVE
Dispositional Greed	Dispositional Greed	DG1	0.815	0.89	0.89	0.53
		DG2	0.840			
		DG3	0.667			
		DG4	0.780			
		DG5	0.748			
		DG6	0.686			
		DG7	0.496			
Well-being	Life Satisfaction	LS1	0.799	0.88	0.88	0.61
		LS2	0.914			
		LS3	0.946			
		LS4	0.693			
		LS5	0.449			
	Psychological Richness	PR1	0.691	0.95	0.95	0.63
		PR2	0.832			
		PR3	0.612			
		PR4	0.852			
		PR5	0.897			
		PR6	0.888			
		PR7	0.814			
		PR8	0.742			
		PR9	0.826			
		PR10	0.788			
		PR11	0.748			
		PR12	0.756			
	Presence of Meaning	MP1	0.884	0.93	0.93	0.76
		MP2	0.868			
		MP3	0.882			
		MP4	0.862			
	Search for Meaning	MS6	0.826	0.94	0.94	0.76
		MS7	0.772			
		MS8	0.859			
		MS9	0.946			
		MS10	0.942			

Note. *N* = 586. All factor loadings are standardized and statistically significant at *p* < 0.001. AVE = average variance extracted; CR = composite reliability. DG = Dispositional Greed; LS = Life Satisfaction; PR = Psychological Richness; MP = Presence of Meaning; MS = Search for Meanin. Cronbach’s alpha for presence of meaning = 0.93, computed after removing Item 5.

**Table 4:** Model Fit Comparison for Latent Profile Analysis of the Greed Factor.

Model	Classes	LogLik	AIC	BIC	SSABIC	Entropy	Smallestclass (%)	BLRTp	Accuracy
EII	2	−870.74	1749.49	1766.98	1754.28	0.167	36.3	1.000	69.6
EII	3	−856.91	1725.83	1752.07	1733.02	0.740	3.9	0.002	87.1
EII	4	−854.49	1724.99	1759.97	1734.57	0.672	3.9	0.066	83.3
VII	2	−870.49	1750.97	1772.84	1756.97	0.1471	34.8	0.882	67.3
**VII**	**3**	−**834.42**	**1684.83**	**1719.82**	**1694.42**	**0.616**	**6.7**	**0.002**	**80.8**

Note. *N* = 586. EII = equal variance and covariance; VII = varying variance and equal covariance. The selected best-fitting model is indicated in bold. BLRT = bootstrap likelihood ratio test; AIC = Akaike information criterion; BIC = Bayesian information criterion; SABIC = sample-size adjusted BIC.

**Table 5:** Profile Characteristics: Model-Based Estimates and Observed Descriptive Statistics for the Three-Class Model (VII-3).

Class	Label	N	%	M(cond)	SD(cond)	Variance	M(obs)	SD(obs)	Min	Max
1	Low-Greed Profile	39	6.7	−0.870	1.135	1.288	−2.165	0.556	−3.418	−1.591
2	Average-Greed Profile	492	84.0	−0.010	0.795	0.632	−0.043	0.742	−1.579	1.653
3	High-Greed Profile	55	9.4	1.917	0.139	0.019	1.920	0.132	1.671	2.098

Note: M(cond)and SD(cond) = conditional mean and standard deviation estimated from the latent profile model; M(obs) and SD(obs) = observed mean and standard deviation based on posterior class membership.

**Table 6:** Average Latent Class Probabilities for Most Likely Class Membership (Row) by Latent Class (Column).

Assigned\Latent	1. Low-Greed Profile	2. Average-Greed Profile	3. High-Greed Profile
1. Low-Greed Profile	**0.724**	0.276	0.000
2. Average-Greed Profile	0.153	**0.843**	0.005
3. High-Greed Profile	0.019	0.124	**0.857**

Note: Diagonal values (in bold) represent classification accuracy for each class. Overall average accuracy = 80.8%. Class 1 = 72.4%; Class 2 = 84.3%; Class 3 = 85.7%.

**Table 7:** BCH-Corrected Distal Outcome Means and Wald Tests by Dispositional Greed Profile.

Distal Outcome	Low-Greed M (SE)	Average-Greed M (SE)	High-Greed M (SE)	Wald *F*	df1, df2	*p*
Life Satisfaction	−0.15 (0.08)	0.07 (0.05)	0.09 (0.21)	2.80	2, 583	0.062
Psychological Richness	−0.19 (0.07)	0.07 (0.04)	0.28 (0.16)	6.68	2, 583	0.001 **
Presence of Meaning	−0.18 (0.09)	0.07 (0.05)	0.30 (0.22)	3.86	2, 583	0.022 *
Search for Meaning	−0.28 (0.09)	0.06 (0.05)	0.6 (0.16)	12.57	2, 583	<0.001 ***

Note. M = BCH-corrected mean. SE = standard error. Wald F = omnibus Wald *F*-test. df = degrees of freedom. *N* = 586. Low-Greed = Class 1; Average-Greed = Class 2; High-Greed = Class 3. * *p* < 0.05. ** *p* < 0.01. *** *p* < 0.001.

**Table 8:** Pairwise Comparisons and Effect Sizes for BCH-Corrected Means Across Greed Profiles.

Distal Outcome	Comparison	Mean Diff	SE	95% CI	*t*(583)	*p*	*d*	Magnitude
Life Satisfaction	C1 vs. C2	−0.22	0.09	[−0.40, −0.03]	−2.33	0.060	−0.21	Small
	C1 vs. C3	−0.25	0.23	[−0.69, 0.20]	−1.09	0.829	−0.21	Small
	C2 vs. C3	−0.03	0.22	[−0.45, 0.39]	−0.13	1.000	−0.03	Negligible
Psychological Richness	C1 vs. C2	−0.26	0.08	[−0.42, −0.10]	−3.20	0.004 **	−0.29	Small
	C1 vs. C3	−0.48	0.17	[−0.81, −0.14]	−2.77	0.017 *	−0.48	Small
	C2 vs. C3	−0.22	0.16	[−0.54, 0.10]	−1.34	0.542	−0.24	Small
Presence of Meaning	C1 vs. C2	−0.25	0.10	[−0.45, −0.05]	−2.45	0.044 *	−0.22	Small
	C1 vs. C3	−0.48	0.24	[−0.95, −0.01]	−2.00	0.137	−0.37	Small
	C2 vs. C3	−0.23	0.23	[−0.68, 0.22]	−1.00	0.960	−0.19	Negligible
Search for Meaning	C1 vs. C2	−0.34	0.10	[−0.53, −0.15]	−3.44	0.002 **	−0.32	Small
	C1 vs. C3	−0.88	0.19	[−1.24, −0.52]	−4.75	<0.001 ***	−0.75	Medium
	C2 vs. C3	−0.54	0.17	[−0.87, −0.21]	−3.17	0.005 **	−0.54	Medium

Note. Mean Diff = mean difference. SE = standard error. CI = confidence interval. All *p*-values and confidence intervals are Bonferroni-adjusted for multiple comparisons within each distal outcome (three pairwise comparisons per outcome; adjusted α = 0.05/3). Low-Greed = Class 1 (C1); Average-Greed = Class 2 (C2); High-Greed = Class 3 (C3). Cohen’s *d* calculated using pooled standard deviation with class-specific variances. Effect size magnitudes: Negligible (|*d*| < 0.2), Small (0.2 ≤ |*d*| < 0.5), Medium (0.5 ≤ |*d*| < 0.8), Large (|*d*| ≥ 0.8). 95% CI = 95% confidence interval. * *p* < 0.05. ** *p* < 0.01. *** *p* < 0.001.

## Data Availability

Dataset available on request from the authors.

## Supplementary Materials

The following supporting information can be downloaded at: https://www.mdpi.com/article/10.3390/bs16081286/s1, including the compressed file “Supplementary materials R codes.zip” (BCH.R, CFA_final_run (exclude_MP_item5).R, CFA_first_run(including_MP_item5).R, Greed_LPA.R, LPA_VII_classify.R).
